# Clinical outcomes of hemodialysis patients in a public-private partnership care framework in Italy: a retrospective cohort study

**DOI:** 10.1186/s12882-019-1224-2

**Published:** 2019-02-01

**Authors:** L. A. Pedrini, A. C. Winter, F. Cerino, A. M. Zawada, M. Garbelli, A. Feuersenger, A. Feliciani, P. Ruggiero, S. Civardi, C. Amato, B. Canaud, S. Stuard, A. Karch, A. Gauly

**Affiliations:** 1 Department of Nephrology and DialysisNephroCare-ASST Bergamo-Est, Seriate, Italy; 2 Fresenius Medical Care Deutschland GmbH, Bad Homburg, Germany; 3 NephroCare S.p.A., Naples, Italy; 4 Fresenius Medical Care Italia S.p.A, Palazzo Pignano, Italy

**Keywords:** Hemodialysis, Hemodiafiltration, Survival, Outcome, Hospitalization, Outsourcing, Public private partnership

## Abstract

**Background:**

Innovative care models such as public-private partnerships (PPPs) may help meet the challenge of providing cost-effective high-quality care for the steadily growing and complex chronic kidney disease population since they combine the expertise and efficiency of a specialized dialysis provider with the population care approach of a public entity. We report the five-years main clinical outcomes of a population of patients treated on hemodialysis within a PPP-care model in Italy.

**Methods:**

This descriptive retrospective cohort study consisted of all consecutive hemodialysis patients treated in the NephroCare-operated Nephrology and Dialysis unit of the Seriate Hospital in 2012–2016, which exercises a PPP-care model. Clinical and treatment information was obtained from the European Clinical Database. Hospitalization outcomes and cumulative all-cause mortality incidences that accounted for competing risks were calculated.

**Results:**

We included 401 hemodialysis patients (197 prevalent and 204 incident patients) in our study. The mean cohort age and age-adjusted Charlson Comorbidity Index were 67.0 years and 6.7, respectively. Patients were treated with online high-volume hemodiafiltration or high-flux hemodialysis. Parameters of treatment efficiency were above the recommended targets throughout the study period. Patients in the PPP experienced benefits in terms of hospitalization (average number of hospital admissions/patient-year: 0.79 and 1.13 for prevalent and incident patients, respectively; average length of hospitalization: 8.9 days for both groups) and had low cumulative all-cause mortality rates (12 months: 10.6 and 7.8%, 5 years: 42.0 and 35.9%, for prevalent and incident patients, respectively).

**Conclusions:**

Results of our descriptive study suggest that hemodialysis patients treated within a PPP-care model framework received care complying with recommended treatment targets and may benefit in terms of hospitalization and mortality outcomes.

**Electronic supplementary material:**

The online version of this article (10.1186/s12882-019-1224-2) contains supplementary material, which is available to authorized users.

## Background

Recent data show that the worldwide prevalence of chronic kidney disease (CKD) is 10–16% and is rising steadily. This places a significant economic burden on our healthcare systems because although only < 1% of CKD patients progress to end-stage renal disease (ESRD), ESRD associates with high morbidity and mortality. Common comorbidities are cardiovascular disease, diabetes, anemia, and mineral and bone disease. Along with infections, these comorbidities are the main causes of hospital admission in ESRD. This, together with the fact that ESRD patients require dialysis or kidney transplantation, makes ESRD one of the most expensive chronic diseases. Indeed, ESRD treatments account for 5% of the total annual healthcare budgets of countries globally [1–5].

To reduce the impact of ESRD on patients and healthcare systems, it is essential to identify people with impaired kidney function early, prevent CKD progression, and provide ESRD patients with evidence-based healthcare. Innovative care models that alleviate the burden on public healthcare systems are also needed. These may include public-private partnerships (PPPs), which are cooperative alliances between public and private healthcare providers. Only few studies, mainly conducted in Asian countries, have evaluated this care model in ESRD with promising results [6–8]. Clearly, however, further studies on the impact of PPP-care models on ESRD outcomes are needed.

Like many countries, including other European countries, Italy must provide the growing ESRD-patient population with excellent care while containing public healthcare expenditure. Of the 70,000 Italian ESRD patients, 46,000 require chronic hemodialysis. The majority are treated in the 332 public Nephrology and Dialysis units of public hospitals [9, 10]. The remaining 25% of patients are treated in privately-operated clinics, to which some Italian regions have partially outsourced hemodialysis treatment.

In 2010, the Seriate Hospital formed a PPP with NephroCare Italy, which is a subsidiary of Fresenius Medical Care, a private dialysis provider that operates > 3600 dialysis centers worldwide. The purpose of the PPP is to coordinate the clinical activities of the hospital’s Nephrology and Dialysis unit with the clinical activities of other hospital departments and primary-care units of the public healthcare system. To achieve this, NephroCare Italy is in charge of the hospital’s Nephrology Department, which includes an inpatient ward, a central dialysis unit, and five dialysis satellite units. This framework not only treats hemodialysis patients, it also provides patients from other departments with nephrology consultations [11]. The clinical and administrative activities of this framework are monitored and periodically audited by the public health authorities to ensure high-quality care and adherence to public health accreditation standards.

Since the inception of this framework, the patient clinical outcomes have been continuously recorded via European Clinical Database (EuCliD) [12]. These data were used in this retrospective cohort study to describe clinical characteristics, treatment parameters and main clinical outcomes (mortality and hospitalization outcomes) of this cohort of dialysis patients over 5 years, with reference to data reported in regional, national, and international registries.

## Methods

### Participants

All consecutive adult patients who were treated between 1 January, 2012 and 31 December, 2016 in the NephroCare-operated dialysis unit in Bolognini Hospital, Seriate (Lombardy, Italy) and who provided written consent for use of their clinical information for research purposes were identified. Although the PPP-model was initiated in 2010, our analyses focus on the study period 2012–2016 to ensure full implementation of NephroCares’ structures, processes and treatment guidelines and to reduce possible carryover effects from previous treatment policies.

Patients were excluded if they were < 18 years old at the study start (1 January, 2012) or renal-replacement therapy initiation date or key treatment variables were missing. No other patients, e.g. patients with distinct comorbidities, were excluded. Prevalent and incident patients were grouped separately given that they may differ in their baseline characteristics and as well as in their mortality risks and trajectories (Additional file 1: Figure S1). Prevalent patients were those whose renal-replacement therapy initiation date was on or before 1 October, 2011; thus, all were treated for ≥3 months before the study started. Incident patients were those whose renal-replacement therapy initiation date was after 1 October, 2011 and who received their first hemodialysis treatment in the Seriate dialysis unit within 30 days of their renal-replacement therapy initiation date. Thus, the prevalent-patient index date was the study start date (1 January, 2012). The incident-patient index date was the date of first treatment in the Seriate dialysis unit (i.e., any time between 1 January, 2012 and 31 December, 2016). All patients were followed from their index date until the date of death, kidney transplantation, change in dialysis center, change in treatment (including change to peritoneal dialysis, treatment stop, and spontaneous recovery), other termination reasons, or the end of the study period.

### Data source

Relevant variables were retrieved from EuCliD. This clinical information system has been established within the framework of Fresenius Medical Care’s Quality Improvement and Management Programs in NephroCare Clinics worldwide. It captures routinely collected patient data, including demographics, medical history, laboratory values, prescription data, treatment variables, and clinical outcomes. Disease information is classified according to International Classification of Diseases, version 10.

### Patient and treatment characteristics

Patient demographics at the index date and relevant comorbidities throughout follow-up were extracted. The coding algorithm proposed by Quan et al. was used to classify comorbidities and calculate the Charlson Comorbidity Index (CCI) and the age-adjusted CCI [13, 14]. Patients were categorized according to the treatment modality (high-flux hemodialysis [hf-HD], online post-dilution hemodiafiltration [post-HDF], or online mixed-dilution hemodiafiltration [mixed-HDF]) and vascular-access type (arteriovenous fistula, permanent or temporary vascular catheter, and graft) that were used most commonly in the first 6 follow-up months. Continuous treatment variables that reflected dialysis dose and duration in the first 6 follow-up months were averaged. They were effective-treatment time, blood-flow rate, convective volume, substitution volume, percentage of total substitution volume infused in post-dilution modality (for mixed-HDF), online-clearance monitoring Kt/V, dry body weight, average overhydration, and relative overhydration.

Average laboratory values for iron-, calcium/phosphate-, and lipid-metabolism and inflammation status during the first 6 follow-up months were also calculated.

The quality of dialysis treatment over time was assessed by averaging key laboratory and dialysis-adequacy variables of patients who were treated in the unit in December of each follow-up year. Thus, patients lost to follow-up because of transplantation, death, or other reasons were excluded from this analysis.

### Clinical outcomes

Hospitalization outcomes related to all incident hospital admissions during follow-up. They were total hospital-admission number, average hospital-admission number/patient, average hospital-admission number/patient-year, and average hospital stay. Hospital admissions that occurred before the study index date and resulted in patients still being treated in the hospital at the index date were not included in these analyses.

Mortality information, including the date of death during follow-up, was obtained from EuCliD. All-cause mortality was used to calculate cumulative mortality incidence.

### Statistical analyses

Patient and treatment characteristics were expressed as mean ± standard deviation, median (interquartile ranges), or number (%). Hospitalization outcomes were averaged. Cumulative all-cause mortality incidence every 6 months was calculated, taking into account competing risks. The cumulative all-cause incidence function-effect estimate is the sum of mortality incidences up to the follow-up time point t_j_ and can be interpreted as the probability of dying up to time t_j_, accounting for competing risks. Competing risks are intervening events that preclude the onset of the event of interest or modify the probability of observing it [15]. Kidney transplantation, treatment stop, change to peritoneal dialysis, and spontaneous recovery were defined as competing risks. Patients who transferred to another dialysis center or were discharged for other reasons were censored at the date of transfer or discharge.

## Results

Of the 451 patients who underwent dialysis in the NephroCare-operated unit in the Seriate Hospital in 2012–2016, 50 were excluded because they were < 18 years old at study start (*n* = 1) and/or renal-replacement therapy initiation date and/or key treatment variables were missing (*n* = 15 and 38, respectively). Of the remaining 401 patients, 197 and 204 were prevalent and incident patients, respectively.

### Patient characteristics

At the index date, prevalent and incident patients were on average 66.8 and 67.2 years old, respectively. The mean dialysis vintage of prevalent patients was 70.3 months. Compared to prevalent patients, incident patients had higher body-mass indices (calculated with dry-weight estimations). The groups were similar in terms of gender and comorbidity frequencies apart from diabetes mellitus, which was more common in incident patients (Table 1). In line, the percentage of diabetes mellitus as cause of renal disease was higher in incident than prevalent patients (Additional file 2: Table S1). Most prevalent and incident patients received erythropoiesis-stimulating drugs (90.4 and 87.3%, respectively) and intravenous iron (94.9 and 87.7%, respectively) at least once during follow-up (Additional file 3: Table S2).

**Table 1:** Patient characteristics^b^

Characteristics	Prevalent patients	Incident patients	All patients
	*n* = 197	*n* = 204	*n* = 401
Age, years	66.8 (14.5)	67.2 (12.9)	67.0 (13.7)
Gender			
Female	73 (37.1)	64 (31.4)	137 (34.2)
Male	124 (62.9)	140 (68.6)	264 (65.8)
Dry body weight, kg	68.6 (13.9)	74.1 (15.3)	71.4 (14.8)
Body mass index^a^, kg/m^2^	25.1 (4.1)	27.1 (6.2)	26.2 (5.4)
CCI^b^	4.2 (2.1)	4.2 (2.0)	4.2 (2.0)
Age-adjusted CCI^b,c^	6.7 (2.8)	6.7 (2.6)	6.7 (2.7)
Single CCI comorbidities^b^			
Myocardial infarction	24 (12.2)	25 (12.3)	49 (12.2)
Congestive heart failure	13 (6.6)	12 (5.9)	25 (6.2)
Peripheral vascular disease	72 (36.5)	74 (36.3)	146 (36.4)
Cerebrovascular disease	29 (14.7)	31 (15.2)	60 (15.0)
Dementia	10 (5.1)	8 (3.9)	18 (4.5)
Chronic pulmonary disease	19 (9.6)	21 (10.3)	40 (10.0)
Rheumatic disease	1 (0.5)	1 (0.5)	2 (0.5)
Peptic ulcer disease	5 (2.5)	10 (4.9)	15 (3.7)
Mild liver disease	35 (17.8)	11 (5.4)	46 (11.5)
Moderate or severe liver disease	2 (1.0)	2 (1.0)	4 (1.0)
Diabetes with chronic complication	47 (23.9)	74 (36.3)	121 (30.2)
Hemiplegia or paraplegia	1 (0.5)	1 (0.5)	2 (0.5)
Renal disease	197 (100.0)	204 (100.0)	401 (100.0)
Any malignancy (except malignant skin neoplasms)	41 (20.8)	42 (20.6)	83 (20.7)
Metastatic solid tumor	5 (2.5)	2 (1.0)	7 (1.7)
HIV/AIDS	2 (1.0)	1 (0.5)	3 (0.7)
Hypertension^d^	84 (42.6)	63 (30.9)	147 (36.7)
Dialysis vintage^e^	70.3 (74.0)	0.2 (0.2)	34.6 (62.6)

The data were expressed as mean (standard deviation) or number (%), as appropriate

^a^Body mass index is based on dry weight

^b^For CCI and single comorbidities, the numbers are derived from the entire follow-up period; the other variables are baseline parameters

^c^For calculation of age-adjusted CCI one point was added to the CCI for each decade of age over 40 (e.g., 50–59 years, 1 point; 80–89 years, 4 points)

^d^Hypertension was defined as predialysis blood pressure: ≥150/85 mmHg

^e^Dialysis vintage is calculated as the number of months from renal replacement initiation date to index date

*AIDS* acquired immunodeficiency syndrome, *CCI* Charlson Comorbidity Index, *HIV* human immunodeficiency virus

### Treatment characteristics

Most patients (> 97%) were treated three times a week during the first 6 follow-up months. The most frequently applied vascular-access type was arteriovenous fistula, followed by catheter and graft; notably, incident patients had a higher frequency of catheter use than prevalent patients (43.1% vs 15.7%), probably related to the high percentage of “late referral” patients. Hf-HD was the most frequently applied treatment in incident patients, while post-HDF was mostly applied in prevalent patients (Table 2). Prevalent and incident patients received a mean dialysis dose (Kt/V) of > 1.40. Prevalent patients tended to have higher blood-flow and Kt/V and lower dry body weight.

**Table 2:** Treatment characteristics and laboratory variables of the patients in the first 6 months of follow-up

	Prevalent patients	Incident patients	All patients
	*n* = 197	*n* = 204	*n* = 401
Most frequently applied treatment modality^a^			
hf-HD	82 (41.6)	178 (87.3)	260 (64.8)
post- HDF	92 (46.7)	22 (10.8)	114 (28.4)
mixed- HDF	23 (11.7)	4 (2.0)	27 (6.7)
Most frequently applied vascular access type^a^			
AV Fistula	145 (73.6)	115 (56.4)	260 (64.8)
Permanent central vascular catheter	30 (15.2)	57 (27.9)	87 (21.7)
Temporary central vascular catheter	1 (0.5)	31 (15.2)	32 (8.0)
Graft	17 (8.6)	1 (0.5)	18 (4.5)
Not known	4 (2.0)	0 (0)	4 (1.0)
Effective treatment time, min	232.4 (11.7)	237.5 (10.9)	235.0 (11.6)
Blood flow rate, ml/min	354.8 (45.7)	292.7 (52.9)	323.2 (58.4)
Convective volume, L/session			
On post-HDF	27.8 (4.4)	30.5 (11.5)	28.3 (6.4)
On mixed- HDF	42.7 (4.1)	40.2 (3.0)	42.3 (4.0)
Substitution volume, L/session			
On post- HDF	25.7 (4.4)	28.7 (11.8)	26.3 (6.5)
On mixed- HDF	40.3 (4.1)	37.4 (2.9)	39.9 (4.0)
% of total substitution volume infused in post-dilution in mixed-HDF	54 (8)	64 (2)	55 (8)
OCM KtV	1.65 (0.25)	1.54 (0.35)	1.59 (0.31)
Dry body weight, kg	68.1 (13.7)	71.3 (14.7)	69.7 (14.3)
Average overhydration^b^	12.2 (7.1)	12.2 (7.1)	12.2 (7.1)
Relative overhydration, %^c^	10.4 (6.8)	10.5 (6.8)	10.5 (6.8)
Laboratory variables			
Hemoglobin, g/dl	11.2 (0.9)	10.4 (1.0)	10.8 (1.0)
Ferritin, μg/l	402.0 (253.8)	185.1 (199.1)	295.0 (252.7)
Transferrin saturation, %	27.9 (15.5)	21.6 (10.6)	24.8 (13.7)
Albumin, g/dl	3.6 (0.4)	3.5 (0.5)	3.5 (0.4)
Total calcium, mg/dl	9.0 (0.5)	8.9 (0.5)	8.9 (0.5)
Phosphate, mg/dl	4.0 (1.1)	4.3 (1.1)	4.2 (1.1)
Intact Parathormon, pg/ml	183.0 (147.1)	206.8 (158.7)	194.4 (153.1)
C-reactive protein, mg/l^d^	0.8 [0.3; 4.9]	4.0 [1.4; 14.3]	2.1 [0.5; 9.0]
Total cholesterol, mg/dl	161.6 (37.6)	170.6 (42.9)	165.8 (40.4)
HDL cholesterol, mg/dl	43.1 (17.4)	45.1 (14.9)	44.1 (16.3)
LDL cholesterol, mg/dl	86.2 (28.4)	97.2 (34.7)	91.4 (31.9)
Triglycerides, mg/dl	152.7 (70.9)	149.4 (73.4)	151.1 (72.0)

The data were expressed as mean (standard deviation) or number (%), as appropriate except where indicated

^a^The most frequently applied treatment characteristic per patient during the 6-month follow-up period is considered for the calculations

^b^Average overhydration was calculated as (predialysis body weight – normohydration body weight) [kg] / extracellular fluid [L] × 100

^c^Relative overhydration was calculated as (predialysis body weight – postdialysis body weight) [kg] / extracellular fluid [L]

^d^The C-reactive protein values were not normally distributed. Therefore, these data are expressed as median (interquartile ranges)

For all laboratory and treatment parameters, except the number of treatment sessions, multiple measurements during the 6-month follow-up period were averaged for each patient, and the descriptive statistics presented in this table was calculated based on these patient means

*AV* arteriovenous, *HDL* high-density lipoprotein, *hf-HD* high-flux hemodialysis, *LDL* low-density lipoprotein, *mixed-HDF* online mixed-dilution hemodiafiltration, *OCM* online clearance monitor, *post-HDF* online post-dilution hemodiafiltration

Mean hemoglobin values of prevalent and incident patients during the first 6 months were 11.2 and 10.4 g/dl, respectively. Both groups had normal mean iron, calcium/phosphate, and lipid-metabolism values. Incident patients had slightly elevated C-reactive protein levels (Table 2).

Dialysis efficiency during the entire study was assessed by calculating averages of treatment variables and clinical results of patients in the unit in December of each year. The techniques, particularly post-HDF and mixed-HDF performed with high convective volume, exhibited optimal dialysis efficiency at all time points, as indicated by mean Kt/V values that exceeded the recommended limits of the guidelines, and good control of anemia and nutritional status, secondary hyperparathyroidism, and phosphate and potassium values (Table 3).

**Table 3:** Dialysis performance indicators during the study period

	2012			2013			2014			2015			2016		
	hf-HD *n* = 75	Mixed-HDF *n* = 47	Post-HDF *n* = 78	hf-HD *n* = 55	Mixed-HDF *n* = 74	Post-HDF *n* = 79	hf-HD *n* = 69	Mixed-HDF *n* = 72	Post-HDF *n* = 76	hf-HD *n* = 91	Mixed-HDF *n* = 71	Post-HDF *n* = 67	hf-HD *n* = 81	Mixed-HDF n = 69	Post-HDF n = 76
Session time, h	3.9 (0.2)	4.0 (0.2)	3.9 (0.2)	4.0 (0.1)	4.0 (0.1)	4.0 (0.1)	4.0 (0.2)	4.0 (0.1)	4.0 (0.1)	3.9 (0.2)	4.0 (0.1)	4.0 (0.1)	4.0 (0.1)	4.0 (0.1)	4.0 (0.1)
Blood flow rate, ml/min	327 (59)	387 (22)	383 (32)	334 (44)	392 (22)	388 (25)	320 (46)	387 (28)	378 (31)	343 (39)	382 (28)	376 (31)	348 (36)	376 (28)	385 (24)
OCM KtV	1.74 (0.49)	1.96 (0.38)	1.97 (0.39)	1.78 (0.31)	1.92 (0.32)	1.96 (0.35)	1.75 (0.42)	1.89 (0.34)	1.91 (0.31)	1.77 (0.35)	1.89 (0.36)	1.88 (0.32)	1.75 (0.28)	1.90 (0.29)	1.89 (0.35)
Substitution volume, L/session	–	41.3 (4.0)	26.2 (4.7)	–	38.6 (4.1)	25.9 (2.8)	–	39.6 (4.7)	26.4 (2.4)	–	37.8 (3.8)	24.4 (3.4)	–	37.9 (3.9)	27.6 (3.7)
Substitution volume infused in post-dilution, %	–	53 (6)	100 (0)	–	62 (9)	100 (0)	–	63 (6)	100 (0)	–	63 (6)	100 (0)	–	63 (4)	100 (0)
Dry body weight, kg	63.5 (13.4)	71.5 (15.4)	70.5 (13.6)	62.6 (15.0)	72.1 (14.0)	69.7 (13.0)	64.1 (15.8)	72.8 (13.4)	71.1 (15.7)	62.7 (11.9)	71.4 (14.2)	77.4 (14.5)	63.8 (12.6)	72.4 (15.3)	75.5 (14.8)
Hemoglobin, g/dl	11.1 (1.5)	11.4 (1.6)	11.3 (1.1)	11.3 (1.3)	11.5 (1.3)	11.4 (1.4)	11.1 (1.1)	11.5 (1.3)	10.9 (1.2)	11.2 (1.1)	11.5 (1.1)	11.0 (1.6)	11.0 (1.1)	11.4 (0.9)	11.2 (1.2)
Intact parathyroid hormone, pg/ml	169 (162)	163 (103)	158 (123)	177 (163)	194 (166)	174 (164)	175 (146)	232 (193)	200 (143)	204 (156)	245 (168)	215 (155)	177 (144)	202 (160)	222 (128)
Phosphate, mg/dl	3.9 (1.3)	4.1 (1.0)	4.1 (1.1)	3.7 (1.1)	3.7 (1.0)	4.1 (1.2)	4.1 (1.2)	3.6 (1.0)	3.9 (0.9)	4.0 (1.1)	3.9 (1.2)	4.2 (1.1)	3.9 (1.1)	4.0 (1.1)	4.3 (1.1)
Potassium, mmol/l	4.8 (0.6)	5.0 (0.6)	4.9 (0.8)	4.8 (0.7)	4.9 (0.6)	4.9 (0.6)	5.0 (0.6)	4.8 (0.5)	4.8 (0.5)	4.7 (0.6)	5.0 (0.6)	4.9 (0.6)	4.8 (0.6)	4.9 (0.6)	4.8 (0.6)
nPCR, g/kg/day	1.13 (0.47)	1.12 (0.31)	1.13 (0.44)	1.05 (0.28)	1.14 (0.31)	1.11 (0.26)	1.04 (0.26)	1.04 (0.25)	1.11 (0.21)	1.03 (0.24)	1.08 (0.21)	1.11 (0.22)	1.07 (0.30)	1.01 (0.23)	1.03 (0.25)

The data were expressed as mean (standard deviation)

Patients included in this part of the analyses are all patients who received treatment in Seriate Dialysis Units during December of the respective year

*OCM* online clearance monitor, n*PCR* normalized protein catabolic rate

### Clinical outcomes

Mean follow-up of prevalent and incident patients were 3.17 and 1.80 years, respectively. Prevalent and incident patients had 491 and 415 hospital admissions during this period, respectively. Thus, on average, they had 2.49 and 2.03 hospital-admissions/patient and 0.79 and 1.13 hospital-admissions/patient-year, respectively. The groups had the same average hospital stay (8.9 days) (Table 4).

**Table 4:** Hospitalization outcomes of patients throughout the study period

	Prevalent patients	Incident patients	All patients
	*n* = 197	*n* = 204	*n* = 401
Mean (min; max) follow-up time	3.17 (0.01; 5.00)	1.80 (0.01; 4.78)	2.47 (0.01; 5.00)
Total number of hospital admissions	491	415	906
Average number of hospital admissions per patient^a^	2.49	2.03	2.26
Average number of hospital admissions per patient year	0.79	1.13	0.91
Average length of hospital stay, days (SD)	8.9 (11.0)^b^	8.9 (11.4)	8.9 (11.2)

^a^Numbers relate to the complete follow-up time

^b^One extreme value (hospital stay of 367 days) was excluded from the analysis

**Fig. 1:**
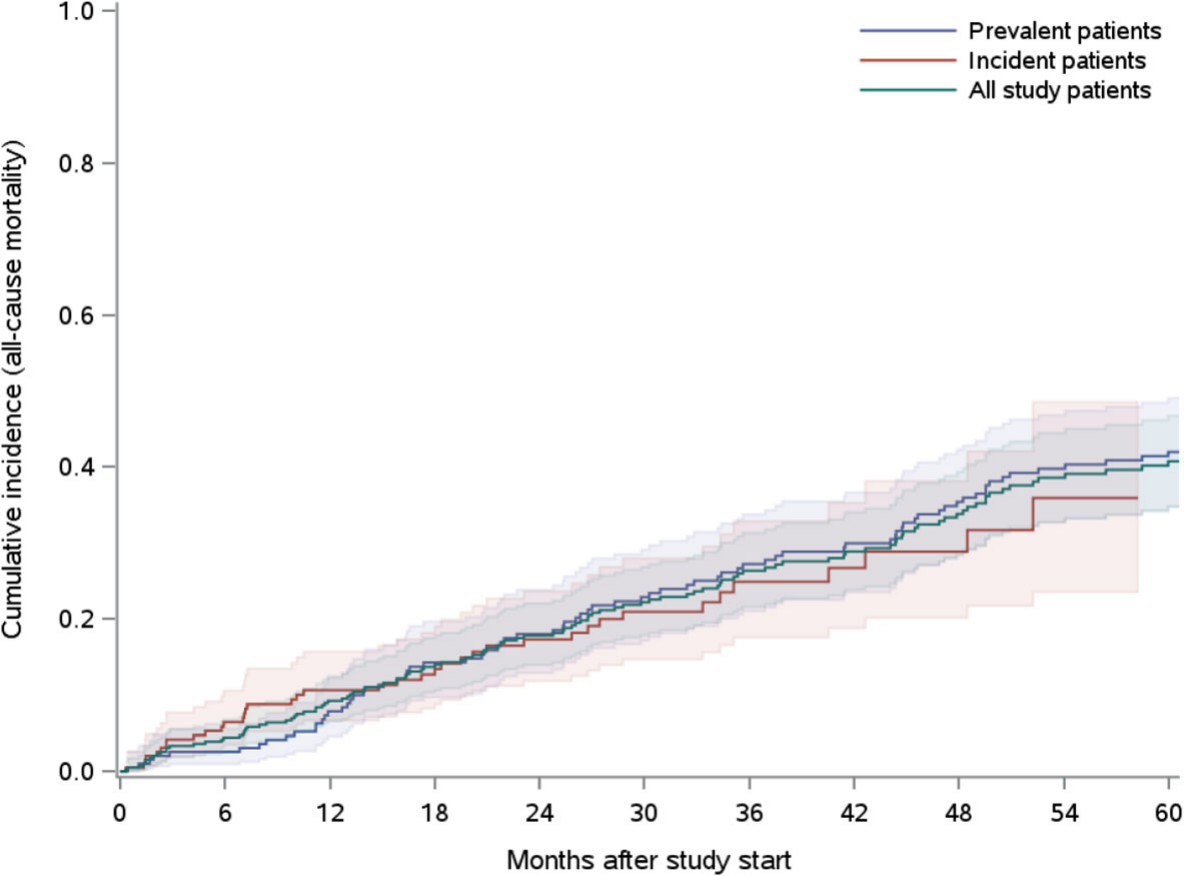
Plot showing the cumulative all-cause mortality incidences of prevalent (blue) and incident (red) patients as well as of the total study population (green)

In total, 117 (78 prevalent and 39 incident) patients died. During follow-up, 55 patients developed a pre-specified competing-risk event, namely, kidney transplantation (*n* = 43), treatment stop (*n* = 8), change to peritoneal dialysis (*n* = 1), and spontaneous recovery (*n* = 3). Eleven and fifteen patients were transferred to another dialysis center or were discharged for other reasons, and were therefore censored at the date of transfer/discharge. In the first 12 follow-up months, incident patients had higher probabilities of dying (10.6%) than prevalent patients (7.8%). Between 12 and 24 months, the cumulative all-cause mortality incidences of the two groups converged: 2-year incidences were 18.0 and 17.3% for prevalent and incident patients, respectively. During the rest of follow-up, prevalent patients tended to have higher probabilities of dying. At the end of the 5-year follow-up, prevalent and incident patients had cumulative all-cause mortality incidences of 42.0 and 35.9%, respectively (Table 5, Fig. 1, Additional file 4: Table S3).

**Table 5:** Cumulative all-cause mortality incidences of the patients every 6 months throughout the study period

	Prevalent patients			Incident patients			All patients		
FU [month]	CIF	Lower 95% CI	Upper 95% CI	CIF	Lower 95% CI	Upper 95% CI	CIF	Lower 95% CI	Upper 95% CI
6	0.026	0.010	0.055	0.064	0.035	0.106	0.044	0.027	0.068
12	0.078	0.046	0.122	0.106	0.066	0.157	0.092	0.065	0.125
18	0.143	0.097	0.197	0.134	0.088	0.191	0.140	0.106	0.178
24	0.180	0.129	0.239	0.173	0.118	0.237	0.178	0.140	0.221
30	0.229	0.172	0.291	0.210	0.147	0.280	0.222	0.179	0.268
36	0.272	0.210	0.338	0.249	0.176	0.329	0.264	0.216	0.313
42	0.300	0.235	0.367	0.267	0.188	0.353	0.289	0.239	0.341
48	0.354	0.286	0.423	0.289	0.202	0.382	0.338	0.284	0.394
54	0.398	0.327	0.468	0.359	0.235	0.485	0.386	0.328	0.444
60	0.420	0.348	0.490	0.359	0.235	0.485	0.408	0.347	0.467

*CIF* cumulative incidence function estimate, *CI* confidence interval, *FU* follow-up

## Discussion

The ever-growing prevalence of ESRD causes a huge burden on national health-care systems, which face the challenge of addressing the medical needs for this complex patient population while containing health care costs. Innovative care models, such as PPPs, may be a promising approach to address these challenges, since they combine the expertise and efficiency of a specialized dialysis provider with the population care approach of a public entity. Only a few studies evaluating these care models have been published.

The PPP between the Seriate Hospital and NephroCare guarantees since 2010 the continuous management of the nephrology and dialysis activities of the public Hospital, which covers 380.000 inhabitants and cares (by December 2017) for over 700 CKD out-patients, 250 patients on chronic dialysis, and over 400 patients/year who are hospitalized for acute and chronic renal diseases.

The present retrospective study on this population showed that in 2012–2016 incident hemodialysis patients were more likely to die in the first 12 months after treatment initiation (10.6%) than prevalent patients in the 12 months after study inclusion (7.8%). The 2- and 3-year cumulative all-cause mortality rates were similar but higher in prevalent patients at the end of 5 years (42.0% versus 35.9%).

Mortality rates are important clinical outcomes for patients, payers, and practitioners. To routinely capture and monitor these outcomes, regional, national, and international ESRD registries have been established worldwide. Their data show that mortality in dialysis patients treated in Lombardy has risen steadily from 11.5% in 1992 to 15.9% in 2014 [16]. Similarly, mortality in other Italian regions has risen from 11.7% in 2009 to 18.8% in 2014 [16]. The pooled data from 12 European countries show that their 1-year mortality rates in 2006–2010 and 2009–2013 were 17.8 and 16.7%, respectively [17]. The latter registry also showed that 2-year mortality rates in 2006–2010 and 2009–2013 were 29.6 and 28.1%, respectively, and 58.2% of the 2006–2010 cohort died within the 5-year follow-up period. Thus, our relatively contemporaneous cohort tended to have lower probabilities of dying, both within 1 and 5 years. Also Postorino et al. found that prevalent and incident dialysis patients treated in Italian NephroCare centers in 2010–2011 had a > 20% lower crude risk of dying than patients in the regional hemodialysis registries of Emilia-Romagna and Calabria, but this difference was not observed after adjustment for demographics and relevant comorbidities [18].

Hospitalization also significantly impacts individuals and economies. Thirteen percent of the annual medical costs of Italian dialysis patients relates to hospitalizations [19]. An Italy-wide study showed that in 1999–2002 dialysis patients had a hospital-admission rate of 0.78/patient-year [20], while more recent studies in Lombardy showed higher rates in 2012 and 2015 of 2.12 and 1.98/patient-year, respectively [16]. Similarly, in 2012–2015, American ESRD patients had 1.7–1.8 hospital-admissions/patient-year [21]. By contrast, during a similar time frame (2012–2016), our prevalent and incident patients had hospital-admission rates of 0.79 and 1.13/patient-year, respectively. Moreover, unlike the Italy-wide study in 1999–2002, which reported a mean hospital stay of 11.6 days [20], our patients had mean hospital stays of 8.9 days. Thus, Italian patients treated within the PPP-care framework tended to have fewer hospital admissions and shorter hospital stays.

However, comparisons between studies of mortality and hospitalization estimates are limited by several factors. First, ESRD associates strongly with comorbidities (e.g.*,* diabetes, hypertension, and cardiovascular disease) that themselves increase the risk of hospitalization and mortality. Therefore, when comparing studies and registers, the comorbidity profile of the patient populations must be considered. In our cohort, 30.2, 36.4, 12.2, and 15.0% had diabetes, peripheral vascular disease, myocardial infarction, and cerebrovascular disease, respectively. Moreover, 43% were hypertensive at dialysis treatment onset. Of these, 31% remained hypertensive despite normalization of body-water volumes and drug therapy. In the regional registries of Emilia-Romagna and Calabria in 2010–2011, prevalence of diabetes, peripheral vascular disease, coronary artery disease (CAD), cerebrovascular disease, and hypertension was 21–40%, 13–33%, 14–32%, 9–13%, and 44–79%, respectively [18]. Of note, as no information on myocardial infarction as a single CAD comorbidity was reported in the registries, that comparison must be considered with caution. Nonetheless, this overview suggests that the comorbidity rates of our study population appear to be comparable to those of other regional Italian-registry populations. Moreover, although appearing numerically low, also the mean CCI of our present study population is comparable to previously published data on dialysis patients [22–25]. Also the mean age of our study population is comparable to the mean age of patients included in the registries: Italian registries, 65.2–69.5 years; ERA-EDTA registry (Italy), 68.8 for incident patients and 62.0 years for prevalent patients.

Second, registries can differ in the methods used to estimate mortality rates and define at-risk populations. Unfortunately, most registries do not present their results separately for prevalent and incident patients. Given that these populations may differ substantially in their baseline characteristics as well as in their mortality risks and trajectories we a priori designed our study to differentiate between prevalent and incident patients and to elucidate possible differences. Indeed, in our study, prevalent and incident patients differed in certain patient and treatment characteristics and had a different short- and long-term mortality risk pattern. For example, while a higher proportion of incident patients was treated with hf-HD (41.6% [prevalent] vs 87.3% [incident]), a higher proportion of prevalent patients was treated with post-HDF (46.7% vs 10.8%) or Mixed-HDF (11.7% vs 2.0%). In the light of the results of large internationals trials in recent years suggesting better survival of patients treated with convective techniques [26–29], such a difference in treatment characteristics could also contribute to the observed differences in mortality rates. Beyond that, compared to those patients who newly started dialysis treatment in the clinic (incident patients), prevalent patients have already by design a survival benefit given that they have already survived until the study start date. As each cohort study faces the challenge of patient turn-over during the study period (patients leave dialysis care for various reasons and new patients start dialysis treatment during any time of the study period), the comparability between different studies will be clearly increased when distinguishing between prevalent and incident patients and accounting for each patient’s individual follow-up time.

Although our study is purely descriptive and no causal links can be proved, our results suggest that a public-private dialysis healthcare model (PPP) may provide high quality care. This is of particular importance in the context of the increasing economic burden public health care systems face. In fact, investments of the private party have allowed to renew the structures, dialysis systems, technologies, materials and IT support. This also ensured the completion and composition of the medical and nursing team and its continuing education. Moreover, patient care is performed according to international guidelines and the clinical management has ensured high dialysis efficiency and good clinical and pharmacological control of the main uremic disorders. A guideline-driven clinical governance and quality improvement system continuously tracks the quality of patient care and outcomes, and provides real-time feedback to practitioners and clinicians. Indeed, in the present study we show that most clinical and treatment variables of our patients were within recommended target ranges. This is particularly important given that a recent study indicates that there is poor guideline-target attainment in Europe [30]. Finally, convective dialysis techniques with highly efficient high-flux membranes, shown to provide survival benefits in dialysis patients [26–29], are widely applied in the unit, including Mixed-HDF, originally designed and implemented in the Seriate dialysis unit [31, 32] and allowing the application of convective treatment also to patients who cannot be treated with Post-HDF due to high hematocrit or inadequate vascular access/blood flow.

In an ongoing debate, some authors report that providers of any profit status can deliver high-quality care efficiently, whereas others argue that profit status can negatively affect care because resources (personnel and dialysis-session duration) must be rationalized to maintain returns [33–38]. However, our extensive and closely monitored 5-year experience in Seriate suggests that if PPP models are organized to meet the public healthcare-facility criteria that are required to achieve accreditation, they can provide expert, efficient, and high-quality patient care independent of profit status.

Our study has several strengths. First, we were able to continuously follow both prevalent and incident dialysis patients in a care setting that was managed under the organization of a PPP-care model. Second, our data encompassed multiple patient and clinical variables that were routinely collected over a long period via an established clinical information system. Third, we accounted for competing risks when calculating cumulative all-cause mortality incidences.

The following limitations should be considered when interpreting our findings. Our study is descriptive; therefore, we cannot draw any conclusions regarding cause or effect. Given that some patients had missing information on renal-replacement-therapy initiation dates or key treatment variables we had to exclude those patients from our analysis and thus could not analyze the total dialysis population of the NephroCare-operated unit in Seriate Hospital. Moreover, we did not have access to the original data from other registries and thus could only compare our results to published findings. This lack of access also meant that we could not account for comorbid conditions and to separately compare results for prevalent and incident patients to published results.

## Conclusions

Results of our descriptive study suggest that hemodialysis patients treated within a PPP-care model framework received care complying with recommended treatment targets and may benefit in terms of hospitalization and mortality outcomes.

## Additional files

Additional file 1: **Figure S1.** Study design and definition of prevalent and incident patients. This figure illustrates the design of the study and visualizes how prevalent and incident patients were defined. The renal-replacement therapy initiation date was before 1 October, 2011 for prevalent patients and between 1 October, 2011 and 31 December, 2016 for incident patients. The study index date was the study start date (1 January, 2012) for prevalent patients and the date of first treatment in the Seriate dialysis unit (i.e., any time between 1 January, 2012 and 31 December, 2016) for incident patients. (PDF 8 kb)

Additional file 2: **Table S1.** Cause of renal disease of the study patients. This table provides information about the number and percentages of underling renal disease of the study participants. (PDF 17 kb)

Additional file 3: **Table S2.** Use of medication at least once during the study period*.* This table provides information about the medication use in our study population during the study period. (PDF 18 kb)

Additional file 4: **Table S3.** Cumulative numbers of deaths, competing events and censored events in prevalent and incident patients every 6 months throughout the study period. This table provides additional information to Table 5 and Fig. 1. (PDF 24 kb)

## Abbreviations

ACE: Angiotensin converting enzyme
AIDS: Acquired immunodeficiency syndrome
AV: Arteriovenous
CCI: Charlson comorbidity index
CI: Confidence interval
CIF: Cumulative incidence function estimate
CKD: Chronic kidney disease
ESRD: End-stage renal disease
EuCliD: European clinical database
FU: Follow-up
HDL: High-density lipoprotein
hf-HD: High-flux hemodialysis
HIV: Human immunodeficiency virus
IT: Information technology
LDL: Low-density lipoprotein
mixed-HDF: online mixed-dilution hemodiafiltration
nPCR: normalized protein catabolic rate
OCM: Online clearance monitor
post-HDF: online post-dilution hemodiafiltration
PPPs: Public-private partnerships
