# Supplementary material for: Clinical outcomes of hemodialysis patients in a public-private partnership care framework in Italy: a retrospective cohort study
Source: BMC Nephrol. 2019 Feb 1;20:35. doi: 10.1186/s12882-019-1224-2 (PMC6359808; doi:10.1186/s12882-019-1224-2)
Supplement: Supplementary file 1 — Figure S1. Study design and definition of prevalent and incident patients. This figure illustrates the design of the study and visualizes how prevalent and incident patients were defined. The renal-replacement therapy initiation date was before 1 October, 2011 for prevalent patients and between 1 October, 2011 and 31 December, 2016 for incident patients. The study index date was the study start date (1 January, 2012) for prevalent patients and the date of first treatment in the Seriate dialysis unit (i.e., any time between 1 January, 2012 and 31 December, 2016) for incident patients. (PDF 8 kb) [file 12882_2019_1224_MOESM1_ESM.pdf]

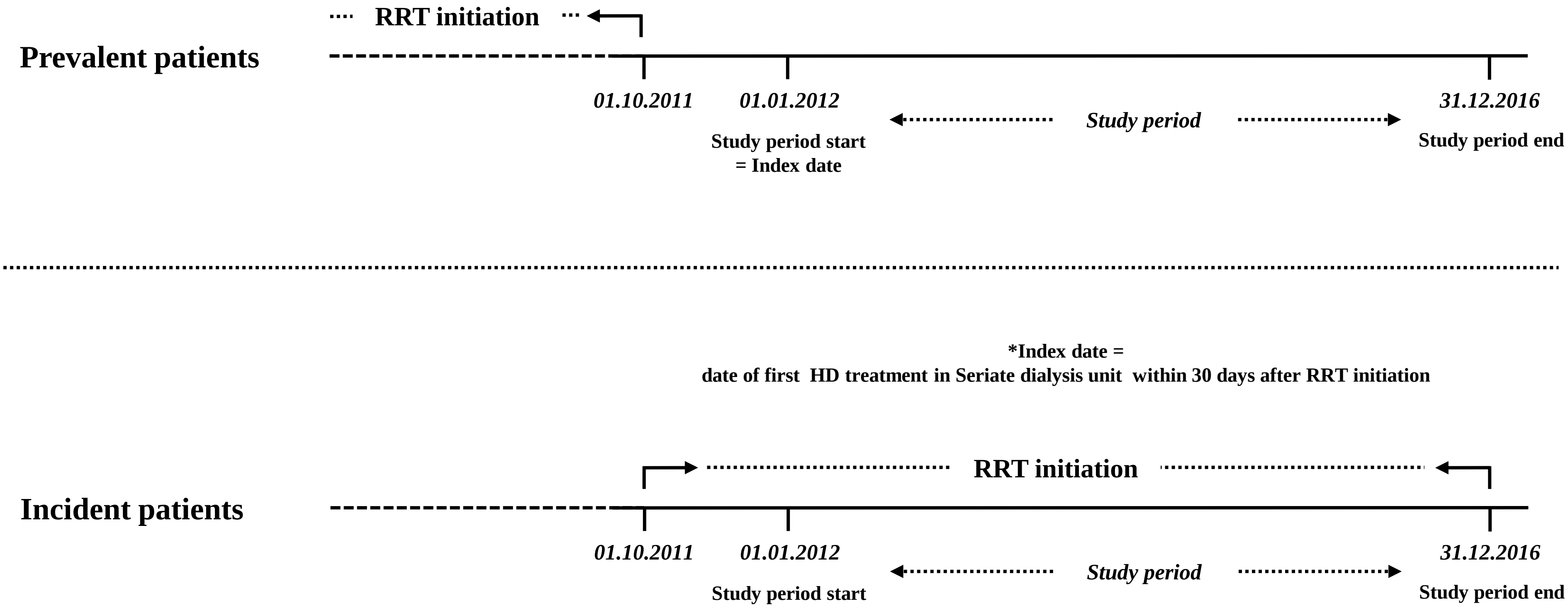
RRT initiation
Prevalent patients
01.10.2011
01.01.2012
31.12.2016
Study period
Study period start
= Index date
Study period end
*Index date =
date of first HD treatment in Seriate dialysis unit within 30 days after RRT initiation
RRT initiation
Incident patients
01.10.2011
01.01.2012
31.12.2016
Study period
Study period start
Study period end

# Figure S1
