# Supplementary material for: Clinical outcomes of hemodialysis patients in a public-private partnership care framework in Italy: a retrospective cohort study
Source: BMC Nephrol. 2019 Feb 1;20:35. doi: 10.1186/s12882-019-1224-2 (PMC6359808; doi:10.1186/s12882-019-1224-2)
Supplement: Supplementary file 2 — Table S1. Cause of renal disease of the study patients. This table provides information about the number and percentages of underling renal disease of the study participants. (PDF 17 kb) [file 12882_2019_1224_MOESM2_ESM.pdf]

Table S1. Cause of renal disease of the study patients

	**Prevalent patients**		**Incident patients**		**All patients**	
	N	*%*	*N*	*%*	*N*	*%*
Diabetes mellitus	22	11.2	45	22.1	67	16.7
Atherosclerosis/Hypertension	16	8.1	14	6.9	30	7.5
Primitive Glomerular Disease	19	9.6	12	5.9	31	7.7
Polycistic/Multicistic kidney disease	14	7.1	10	4.9	24	6.0
Interstitial kidney diseases	17	8.6	10	4.9	27	6.7
Systemic disease (ICD-10 codes M31.-/ E85.-)	4	2.0	3	1.5	7	1.7
Cancer (kidney/ urinary tract)	4	2.0	3	1.5	7	1.7
Other/Unknown etiology[1]	101	51.3	107	52.5	208	51.9
Total	197	100.0	204	100.0	401	100.0

[1]Mainly late referral patients. N = number of patients
