# Supplementary material for: Clinical outcomes of hemodialysis patients in a public-private partnership care framework in Italy: a retrospective cohort study
Source: BMC Nephrol. 2019 Feb 1;20:35. doi: 10.1186/s12882-019-1224-2 (PMC6359808; doi:10.1186/s12882-019-1224-2)
Supplement: Supplementary file 3 — Table S2. Use of medication at least once during the study period. This table provides information about the medication use in our study population during the study period. (PDF 18 kb) [file 12882_2019_1224_MOESM3_ESM.pdf]

Table S2. Use of medication at least once during the study period

Medication	Prevalent patients<br>%	Incident patients<br>%	All patients<br>%
Erythropoesis-stimulating agents	90.4	87.3	88.8
Intravenous iron	94.9	87.7	91.3
Statins	38.1	47.5	42.9
Antihypertensive drugs			
ACE-inhibitors	22.3	11.8	17.0
Beta-blockers	50.8	50.5	50.6
Other	57.9	71.6	64.8
Antidiabetic drugs			
Insulin	16.2	27.5	21.9
Oral anti-diabetic drugs	3.0	6.4	4.7
Phosphate binders			
Calcium acetate/carbonate	0.0	0.0	0.0
Sevelamer	13.7	10.8	12.2
Other phosphate binders	6.1	2.0	4.0
Vitamin D and analogs			
Calcitriol	61.9	58.8	60.3
Paricalcitol	12.7	6.9	9.7
Other	82.7	78.4	80.5
Cinacalcet	15.2	2.5	8.7
Other drugs	69.0	63.7	66.3

ACE Angiotensin Converting Enzyme
