# Supplementary material for: Clinical outcomes of hemodialysis patients in a public-private partnership care framework in Italy: a retrospective cohort study
Source: BMC Nephrol. 2019 Feb 1;20:35. doi: 10.1186/s12882-019-1224-2 (PMC6359808; doi:10.1186/s12882-019-1224-2)
Supplement: Supplementary file 4 — Table S3. Cumulative numbers of deaths, competing events and censored events in prevalent and incident patients every 6 months throughout the study period. This table provides additional information to Table 5 and Fig. 1. (PDF 24 kb) [file 12882_2019_1224_MOESM4_ESM.pdf]

Table S3. Cumulative numbers of deaths, competing events and censored events in prevalent and incident patients every 6 months throughout the study period

Follow-up time [month]	Prevalent patients					Incident patients				
	Patient month	Patients at risk [n]	Deaths [n]	Competing events[1] [n]	Censored events[2] [n]	Patient month	Patients at risk [n]	Deaths [n]	Competing events[1] [n]	Censored events[2] [n]
0	0	197	0	0	0	0	204	0	0	0
6	3.6	184	5	5	4	5.9	156	12	10	27
12	11.9	164	15	11	8	12.0	131	19	12	43
18	17.4	146	27	15	10	18.0	110	23	14	58
24	22.7	136	34	18	10	23.9	88	28	16	73
30	29.8	122	43	22	11	30.0	71	32	17	85
36	35.6	110	51	25	12	35.8	45	35	18	107
42	41.5	104	56	26	12	42.0	30	36	19	120
48	47.9	90	66	30	12	47.2	20	37	21	127
54	52.5	80	74	31	13	52.7	9	39	21	136
60	60.0	74	78	33	13	58.2	1	39	22	143

[1]Kidney transplantation, treatment stop, change to peritoneal dialysis, and spontaneous recovery were defined as competing risks.

[2]Patients who transferred to another dialysis center or were discharged for other reasons were censored at the date of transfer or discharge.
